# Community engagement strategies for antimicrobial resistance control and interventions

**DOI:** 10.1136/bmjph-2026-004874

**Published:** 2026-08-04

**Authors:** Sanjaya Acharya, Thomas J Peto, Abhilasha Karkey, Phaik Yeong Cheah, Bipin Adhikari

**Affiliations:** 1 Academy for Data Science and Global Health, Kathmandu, Nepal; 2 Mahidol-Oxford Tropical Medicine Research Unit, Faculty of Tropical Medicine, Mahidol University, Bangkok, Thailand; 3 Centre for Tropical Medicine and Global Health, Nuffield Department of Medicine, University of Oxford, Oxford, UK; 4 Oxford University Clinical Research Unit, Kathmandu, Nepal

**Keywords:** Community Participation, Public Health, Scoping Review

## Abstract

Antimicrobial resistance (AMR) is a major global health threat spanning biomedical, social and ethical domains. Conventional stewardship and regulatory strategies, often vertical and clinically oriented, frequently overlook community realities and the systemic drivers of antimicrobial use. Focusing on low- and middle-income countries (LMICs), this review blends insights from the literature and authors’ experiential knowledge of working on AMR in LMICs and highlights community engagement as an essential yet underexplored component of AMR control. Community engagement encompasses complementary breadth-based approaches (e.g., awareness-raising activities and population-level communication) and depth-based approaches (e.g., community dialogue, coproduction, shared decision-making and accountability), together forming a ‘diagonal approach’ to developing locally relevant and sustainable AMR interventions. This approach integrates population-level communication with context-specific partnerships involving communities and other One Health stakeholders. Drawing on evidence from community engagement, ethics, health systems and One Health, we examine how integrated engagement approaches across various sectors can address the social and structural determinants of antimicrobial use while complementing stewardship efforts and health system strengthening. We argue that embedding meaningful community engagement within AMR policies and interventions can support equitable, context-sensitive and sustainable AMR governance and strengthen the long-term effectiveness of antimicrobial stewardship.

## Introduction

### Burden of antimicrobial resistance

 Antimicrobial resistance (AMR) refers to the ability of microbes to withstand drugs designed to inhibit or kill them.^1^ In this article, AMR refers primarily to antibacterial resistance.^2^ AMR is one of the most urgent public health crises of the 21st century, weakening modern medicine and imposing major global health and economic burdens.^3^ AMR undermines the treatment of common infections, heightens risks in surgeries, cancer care and childbirth, and could cause up to 10 million deaths annually by 2050 if unaddressed.^4^ The 2019 Global Resistance on Antimicrobial Resistance (GRAM) report estimated that AMR caused 1.27 million deaths and contributed to nearly 5 million globally, disproportionately impacting low- and middle-income countries (LMICs).^4^

In LMICs, weak health systems, limited diagnostic capacity and poor water, sanitation and hygiene (WASH) infrastructure contribute to a high infectious disease burden, often compensated for by excessive antimicrobial use, serving as structural ‘quick-fix’ patches for fragile health systems.^5^ Antimicrobial stewardship monitors antibiotic use across human, animal and environmental sectors, primarily targets supply-side factors. Nonetheless, these approaches often overlook demand-side dynamics such as communities’ treatment seeking behaviours, healthcare network and agricultural and animal husbandry practices, all of which are critical to effective One Health stewardship.^6–8^ Some LMICs have weak and fragmented primary healthcare systems, characterised by shortages of trained health workers, resources (eg, equipment, infrastructure), lack of health insurance, and poor referral pathways, all of which further constrain timely access to appropriate diagnosis and treatment. In these settings, antimicrobial use often compensates for deficiencies within the health systems and is therefore shaped by limited access to quality healthcare rather than by individual knowledge or preferences alone, underscoring the need to address structural health system barriers alongside behavioural interventions.^9^

Unregulated antimicrobial use in agriculture and aquaculture fuels environmental resistance that threatens both animals and humans.^10,11^ The socio-economic impacts are profound: prolonged illness, costly care and higher mortality deepen poverty and inequality.^12^ Consequently, AMR is not only a biomedical issue but also a social and developmental crisis that threatens progress in global health and exacerbates structural poverty and inequity in LMICs.^13,14^

An overlooked driver of AMR is chronic health system underfinancing, as low tax revenues and limited budgets leave per capita health spending far below that in high-income countries.^15^ As a result, shortages of health workers, overstretched primary care and limited diagnostics lead to antimicrobials being used as substitutes for core health services and as low-cost alternatives in the absence of adequate clinical assessment and testing.^16,17^

In many resource-constrained settings, communities rely on informal healthcare providers, community pharmacies and other alternative sources of care because they are affordable, geographically accessible and responsive to immediate healthcare needs. These care-seeking practices often represent pragmatic responses to health system limitations rather than deliberate misuse of antimicrobials. Recognising these structural realities is essential for designing equitable AMR policies and community engagement strategies that address the underlying drivers of antimicrobial use.^18^

AMR also operates across sectors, as environmental stressors such as heavy metal exposure, industrial pollutants, biocides, wastewater and agricultural residues can trigger bacterial mutations and co-select for resistance genes.^19–21^ Resistance traits linked to heavy metal exposure may persist even without antibiotic pressure,^22^ underscoring the need for renewed, multipronged AMR strategies.

### Limitations of current AMR strategies in reaching communities

Traditional AMR responses have generated important advances in stewardship and surveillance but often remain predominantly top-down, with limited opportunities for communities to participate in identifying priorities, shaping interventions or informing implementation. Consequently, they may overlook the social, economic, cultural and health system contexts that influence antimicrobial use in everyday life. For example, individuals with mild illnesses frequently bypass formal healthcare systems, obtaining over-the-counter (OTC) antimicrobials instead.^23–25^ Community engagement should be seen as a way to identify priorities and barriers to appropriate antimicrobial use and to cocreate feasible solutions through dialogue, mutual learning and shared accountability among communities, professionals, policymakers and One Health stakeholders, rather than treating communities as passive recipients of health messages.^26^

Similarly, unlike commercial livestock and aquaculture which may have regulated use of antimicrobials, small-scale farming in LMICs commonly relies on prophylactic antimicrobial use and antimicrobial-containing feed for growth promotion.^27^ The poorly regulated use of pesticides and veterinary antibiotics also contributes to environmental contamination, with studies reporting residues in up to 95% of vegetables in India and Nepal.^28^ Addressing these challenges requires integrated, multisectoral approaches, as interventions targeting isolated components risk producing unintended consequences.^29,30^

A growing body of evidence underscores the need for bottom-up strategies that address the social, cultural, economic and behavioural drivers of antimicrobial use.^16^ For instance, across five African livestock systems, interventions were most effective when informed by local knowledge, attitudes and practices.^31^ A study in Iran demonstrated that without understanding the sociocultural context of antimicrobial behaviours, control efforts were unlikely to succeed.^32^ Effective AMR mitigation thus depends on participatory, community-engaged approaches that are culturally appropriate, contextually relevant and sustainable.^30,33^ Nonetheless, stakeholder-inclusive approaches remain underutilized in AMR management frameworks.^34^ Strengthening community-level strategies is vital to complement programmatic and policy interventions, ensuring harmonisation between programmes and community expectations.^30,33^

### Understanding community engagement for AMR control

Ideally, community engagement should be a collaborative process through which communities identify priorities, co-design interventions and implement locally relevant solutions through partnership, dialogue and shared decision-making with policymakers.^35,36^ It should promote incorporation of insights from all stakeholders including community members, health professionals and policymakers involved in AMR prevention and control. Nonetheless, engagement occurs along the continuum increasing community influence over priority setting, intervention design, implementation and evaluation.^26,33^ In the context of AMR, a phenomenon characterised by biomedical and social complexities, community engagement is not merely a means to reach the target audiences but a critical component to yield meaningful outcomes.^37^ Across the One Health sectors, antimicrobial use within communities is shaped by treatment-seeking behaviours and perceptions of health risks.

These behaviours are shaped by cultural, social, economic and health system characteristics. In many LMICs, antimicrobials are obtained OTC, used suboptimally, shared among family members or discontinued once symptoms subside. For instance, a study in Uganda illustrated that antibiotics were commonly shared within households, and unused medicines were discarded with household waste.^38^ A systematic review of 38 studies from 24 countries found that antibiotics were frequently obtained without prescriptions from community pharmacies, with non-prescription rates of up to 62% (95% CI 53% to 72%) in some LMICs.^39^ While often described as irrational from a biomedical perspective, such practices can be rational within the lived realities of communities, where limited access to healthcare, and economic hardship (e.g., absence of paid sick leave, daily wage work) make early and unsupervised use of antimicrobials a pragmatic strategy for coping with illness.^40^

Despite being central actors in antimicrobial use, communities are frequently excluded from AMR policy design and programme implementation.^41^ Most interventions have prioritised clinical and regulatory measures, giving comparatively little attention to the social, behavioural, and structural dimensions of antimicrobial use at the community level. Evidence from diverse contexts demonstrates the value of participatory engagement. In rural Bangladesh, co-developing a community-based intervention with local stakeholders underscored the importance of inclusive design and adaptation to existing health systems, community structures and sociocultural environments.^42^ In northern Tanzania, community-based education on dose charts and thermometers improved accuracy of antimicrobial use.^43^ In urban Brazil, aligning stewardship initiatives with local values and experiences bridged gaps between technical guidelines and lived realities.^44^

Misconceptions, such as the belief that antimicrobials can treat viral infections, combined with pervasive suboptimal use, underscore the need to understand antimicrobial practices before designing interventions.^45,46^ Similar trends are observed in the animal health sector: in Kenya, 80% of 319 livestock keepers reported using antibiotics, and 58% did so without veterinary advice, heightening AMR risks.^47^ Across nine African countries, engagement initiatives involving community and animal health workers achieved broad population coverage and meaningful shifts in social norms around antimicrobial use, reinforcing their role in fostering sustainable behavioural change.^48^ Comparable outcomes have been documented in Brazil, where integrating community and healthcare perspectives produced tailored, context-appropriate stewardship models.^44^ In sub-Saharan Africa, participatory engagement with livestock farmers corrected misconceptions and promoted responsible antimicrobial practices in agriculture.^41^

Despite these successes, engagement remains underutilized in AMR responses, where efforts often focus on hospital-based stewardship and national policy. A paradigm shift toward viewing communities as active co-producers of knowledge and solutions is urgently needed.^49^ Such inclusive approaches are essential for socially legitimate, contextually grounded and sustainable AMR interventions. This review blends the authors’ experiential knowledge of working in the LMICs and synthesises evidence from ethics, health systems and One Health perspectives to examine how community engagement can strengthen equitable, context-sensitive and sustainable AMR responses. We argue that community engagement should be considered a core component of AMR governance and mitigation that complements antimicrobial stewardship and broader health system strengthening.

## Main text

### Clinical versus public health interventions for control of AMR

In clinical practice, healthcare providers are ethically and professionally obligated to prioritise individual patient outcomes. This may lead to the use of broad-spectrum or last-resort antimicrobials, such as Meropenem or Colistin under diagnostic uncertainty.^50,51^ Such decisions align with the ethical principle of beneficence and, more broadly, the Hippocratic Oath prioritising the patient’s well-being.^52^ However, this individual-centred obligation can conflict with the public health goal of preserving antimicrobial effectiveness for collective benefit.^53,54^

Although clinically justified in most cases, antimicrobial use may inadvertently contribute to overuse and resistance, undermining efforts to promote equity and sustainability in public health.^55,56^ Balancing these tensions requires coordinated interventions that harmonise clinical ethics and stewardship principles.^57^ Developing context-sensitive prescribing guidelines can help clinicians align patient care with broader AMR goals.^56^ Investments in diagnostics can support rational prescribing, while ethics training can strengthen awareness of the societal implications of antimicrobial use.^58^

Addressing AMR also demands accountability across the supply chain, from prescribing to dispensing. Incentives that drive profit-oriented antimicrobial sales should be minimised through regulation, transparency and engagement with health professionals. Regular dialogue among stakeholders can strengthen stewardship by bridging clinical and public health perspectives.^8,30^

### Ethical dilemmas surrounding AMR control and interventions

AMR control involves a series of ethical trade-offs between access, equity and sustainability. The following dilemmas illustrate the competing moral imperatives that shape decision-making across human, animal and environmental health systems.

#### Access–excess axis

A central challenge in AMR governance lies along the access–excess continuum.^55^ In many LMICs, rural and marginalised populations lack access to essential antimicrobials for treatable infections, leading to avoidable morbidity and mortality. A survey from Bangladesh reported frequent OTC sales of antimicrobials, highlighting how poor-quality access and purchasing practices can promote AMR.^59^ Conversely, in urban areas of several LMICs, unregulated sales and self-medication of antimicrobials are key drivers of inappropriate use, demonstrating that AMR is not solely a consequence of limited access.^55,60^ This inequity reflects a deeper public health injustice: where deprivation and excess coexist within the same health system, and across the geographical region. Addressing AMR therefore requires equity-oriented strategies that expand access while promoting responsible antimicrobial use through stewardship, effective regulation and community engagement.^61^ Community engagement that includes the voices of the most disadvantaged can identify the depth and breadth of vulnerability (characterised by poverty, poor access, social, political and cultural marginalisation) affecting the population, thereby informing the development of equitable interventions for the targeted communities.

#### Immediate versus intergenerational impacts

Antimicrobial use presents an ethical tension between immediate clinical benefit and long-term societal harm. While antibiotics save lives, their use and misuse accelerate resistance, diminishing treatment effectiveness for future generations.^55,62^ Because the link between individual antibiotic use and population-level resistance is indirect, the problem often remains abstract for prescribers and patients, particularly where OTC access, empirical prescribing or agricultural antibiotic use are normalised.^25,63^ This tension highlights the need for a shared ethical commitment to preserve antibiotic effectiveness for future generations, similar to collective action on climate change.^64^

#### Misalignment of interests

Divergent stakeholder interests often conflict, generating complex moral and practical challenges.^30^ Individual and institutional interest can undermine collective goals when profit-driven incentives distort responsible antibiotic use. In many LMICs, drug dispensers depend on antimicrobial sales within poorly regulated private sectors.^65^ Such practices, while shaped by livelihood necessity, often occur in contexts where regulatory guidance is ambiguous and governance fragmented.^66^ This raises important questions about accountability, particularly regarding the balance between individual responsibility and systemic failures in regulation and oversight.

Similar ethical tensions arise for medical and veterinary practitioners, who must balance duties to individual patients with responsibilities to the public good. Prescribing may also be shaped by pharmaceutical incentives, blurring professional integrity and stewardship goals.^67,68^ Effective AMR control therefore requires multi-level accountability across individual, professional and institutional levels while aligning economic incentives with ethical stewardship. These ethical complexities shape everyday decisions across behavioural, economic and social contexts.

#### Behavioural drivers and responses

In LMICs, human and animal health stakeholders make antimicrobial use and sales decisions within complex social, cultural and economic contexts.^30^ Patients may demand antibiotics out of fear, habit or the belief that ‘stronger’ drugs guarantee quicker recovery. Clinicians, facing diagnostic uncertainty or patient expectations, may prescribe empirically or prophylactically to avoid perceived treatment failure.^40^

These behaviours reflect structural conditions such as poverty, livelihood insecurity and limited access to health and veterinary services rather than individual choice alone; ignoring these drivers risks counterproductive outcomes.^69^ Disrupting informal practices without viable alternatives may restrict access to care or undermine livelihoods. Recognising these linkages demonstrates how antimicrobial use is tied to structural poverty, requiring coordinated strategies that pair behavioural change with poverty reduction, health system strengthening and sustainable development.

A growing body of evidence demonstrates that the environmental stressors, such as heavy metals, biocides, industrial pollutants and agricultural contaminants, can activate bacterial stress responses and co-select for resistance determinants.

#### Agricultural and veterinary use

The extensive use of antibiotics in agriculture and veterinary medicine, particularly for prevention and growth promotion, poses a major challenge for AMR control. Global livestock antimicrobial use was estimated at 110,777 tonnes in 2019 and is projected to exceed 143,000 tonnes by 2040, largely driven by LMICs.^70^ Antibiotic use in cattle, pigs and chickens alone represents approximately 76,060 tonnes annually, underscoring the heavy reliance on these drugs in food production systems.^71^ While these practices support food security and livelihoods, especially for smallholder farmers, they also drive the emergence of resistant bacteria that are transmissible to humans via food, environmental pathways or direct contact.^72^

While high-income countries enforce strict antibiotic regulations, applying these models in LMICs without contextual adaptation risks harming livelihoods and food security. Many small- and medium-scale farmers rely on antimicrobials to protect vulnerable livestock, and without affordable alternatives such as vaccination, biosecurity or veterinary services, abrupt withdrawal can cause economic loss, deepen poverty and worsen nutrition.^73^ The challenge therefore lies not in eliminating antimicrobial use, but in enabling a socially just and economically feasible transition to safer, sustainable practices.^74^

It is also important to recognise that current global estimates of antimicrobial use in agriculture are likely underestimated due to reliance on self-reported national data, non-standardised surveillance systems, informal markets and unregulated or internet-based sales.^75,76^

### Development of novel antibiotics

The discovery and development of new antibiotics are time- and resource-intensive, contributing to persistent stagnation in the antibiotic pipeline.^77^ Increasing resistance to first-line antibiotics such as amoxicillin and ceftriaxone is making once-treatable infections harder to manage in resource-limited settings. This is compounded by the spread of resistant pathogens, including *Escherichia coli* and *Klebsiella pneumoniae*, which show declining susceptibility to first-line agents such as ampicillin, co-trimoxazole and cephalosporins. In Malawi, a 2020–2024 study found that over 54% of bloodstream and urinary tract infection isolates were resistant to tested antibiotics.^78^ Without stronger engagement from pharmaceutical companies, the development of new antimicrobials remains limited, reinforcing the need to protect existing drugs through coordinated stewardship. Promoting responsible use across human, animal and environmental sectors, in line with the One Health approach, is the most immediate and feasible way to sustain antimicrobial effectiveness while innovation lags.^79^

### Community engagement strategies for AMR control and interventions

Community engagement operates along a spectrum ranging from broad, population-level initiatives to more targeted, participatory approaches.^80^ Based on levels of engagement, approaches can be broadly categorised as breadth-based or depth-based. Rather than representing competing strategies, they are complementary and should be selected according to the objectives, context and stakeholders involved in AMR prevention and control. Engagement embedded in the One Health approach incorporates multilayered and multisectoral strategies.^37^ Those affected include not only patients and households but also farmers, veterinarians, pharmacists, food industry workers and water and sanitation stakeholders. These stakeholders play complementary roles in AMR prevention. Pharmacists and informal drug sellers can promote appropriate dispensing and referral, community health workers can support household-level stewardship and health education, farmers and veterinarians can encourage responsible antimicrobial use in livestock, while schools, local governments and WASH actors can strengthen infection prevention and community mobilisation. Tailoring engagement through stakeholder analysis and sector-specific dialogue can therefore improve the relevance and effectiveness of AMR interventions.^30,33^ The invisible and ‘super-wicked’ nature of AMR further complicates community engagement, requiring approaches that address both the use of antibiotics and their consequences, connecting resistance to the realities of everyday life.^37^ For the purpose of this discussion, we categorise community engagement strategies into two forms: breadth-based approaches and depth-based approaches (online supplemental table S1).^81^

Breadth-based approaches refer to large-scale communication campaigns, policy advocacy and public engagement initiatives that build population-level understanding of AMR and support coordinated stewardship efforts. While they are effective for establishing shared priorities and mobilising action at scale, they are unable to address the diverse social, cultural and structural factors influencing antimicrobial use within individual communities.^82^ For instance, a review of global AMR campaigns found widespread reliance on mass media and public messaging, but with mixed success due to limited contextual adaptation.^82^ Similarly, the WHO Global Action Plan on AMR adopts a breadth-based strategy, using education and media outreach to promote responsible antimicrobial use across diverse populations.^83^

In contrast, streamlined approaches are localised and context-specific, emphasising coproduction, shared decision-making, and meaningful participation.^43^ They involve listening to community perspectives, identifying local priorities, facilitating dialogue and coproducing tailored stewardship interventions with farmers, informal providers and other local stakeholders. Although more time-intensive and resource-intensive, these approaches often strengthen local ownership and accountability, generating interventions that are more relevant, acceptable and sustainable. For example, community conversations in rural Ethiopia improved antimicrobial practices,^46^ while Bangladesh’s Community Dialogue Approach fostered trust and sustained behaviour change through culturally sensitive discussions.^42^

While breadth- and depth-based approaches serve different purposes, their effectiveness depends on how they engage local systems, power relations and pathways to sustainability.^30^ Breadth-based strategies, including policy advocacy, national action plans and media campaigns, help mobilise political will and resources for AMR action. However, their impact may be limited unless, they are adapted to local realities through sustained investment, local leadership and mechanisms that embed engagement within national AMR action plans and routine health systems.^33^ In addition, there may be unintended consequences.

Streamlined approaches help identify the social, cultural and structural factors influencing antimicrobial use, enabling interventions to be coproduced with communities and better aligned with local priorities and limitations.^42^ However, these resource-intensive approaches require sustained funding and institutional support, and their broader impact depends on integrating locally generated knowledge into health system planning and national AMR action plans.

**Figure 1:**
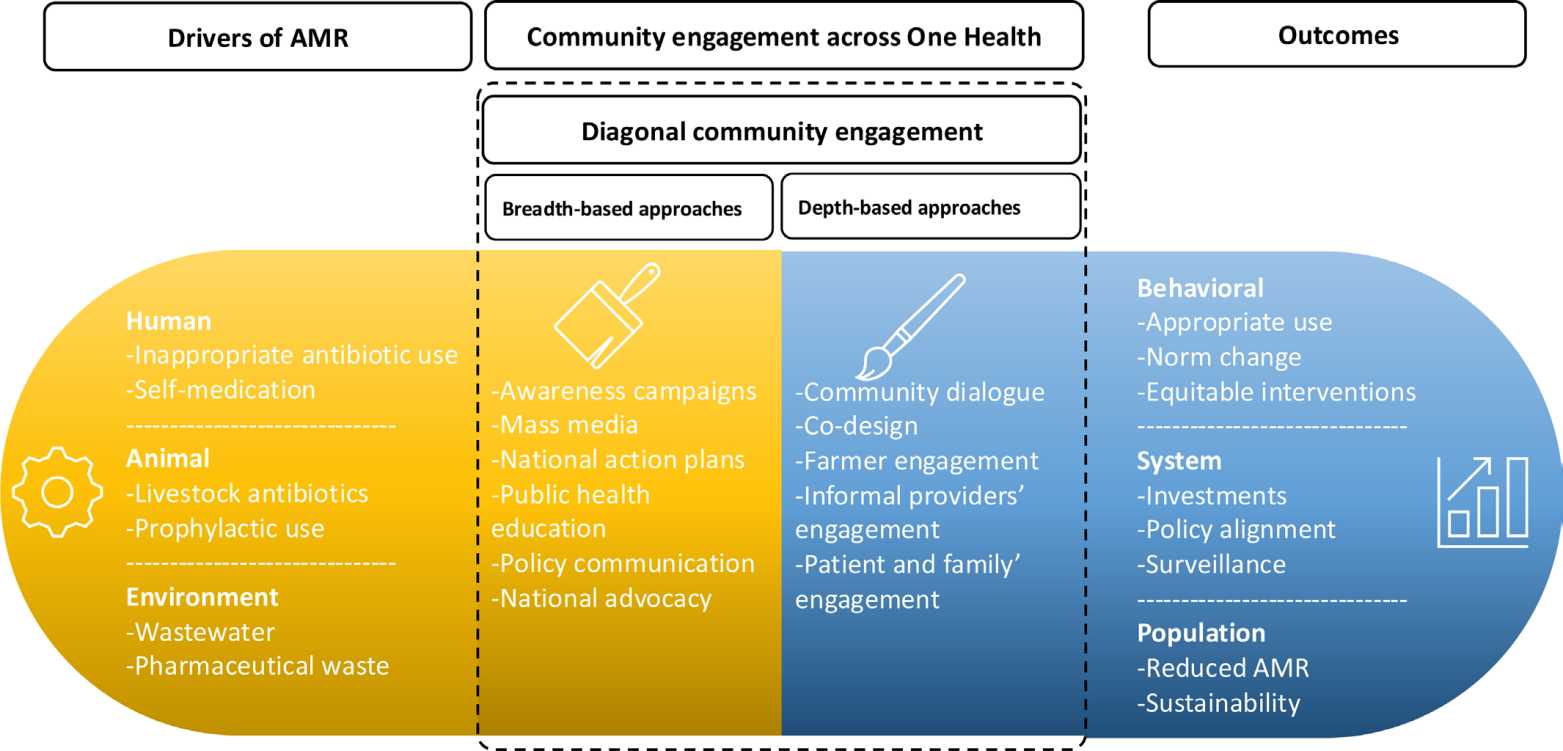
Proposed diagonal community engagement framework illustrating how breadth-based and depth-based approaches across One Health sectors address key drivers of antimicrobial resistance and contribute to behavioural, system and population outcomes.

Neither approach alone is sufficient. Effective AMR control requires integrating breadth-based reach with depth-based participation. Together, they form a ‘diagonal approach’ in which national policies draw on community insights, local initiatives are reinforced by institutional leadership and community engagement is embedded within routine health systems.^84^ In LMICs, integrating community engagement into national AMR action plans has strengthened identification of local needs, improved intervention alignment and enhanced programme ownership.^33,85^ Achieving this integration requires sustained investment by health systems, institutional leadership and mechanisms that enable community priorities to inform national AMR action plans and their implementation. Our proposed diagonal community engagement framework (figure 1) illustrates how breadth-based and depth-based engagement across One Health sectors can support equitable, contextually grounded and sustainable AMR interventions.

Future studies should empirically examine how integrated engagement models operate across human, animal and environmental sectors within a One Health framework. Building on existing evidence, participatory and realist methods could assess what works, for whom and under what conditions in co-developing AMR interventions.^86^ For example, multimethod studies in LMIC settings could identify feasible engagement strategies, assess cross-sector transferability and refine mechanisms that promote sustainability and equity. Such evidence is critical for translating engagement theory into effective, context-sensitive AMR policy and practice.

## Conclusions

Community engagement places people at the centre of AMR responses by enabling them to identify challenges, co-design solutions and implement interventions, helping uncover the drivers of antimicrobial use, build trust, develop locally relevant strategies and minimise unintended consequences. Within the One Health framework, it bridges ethical and practical tensions across sectors through shared understanding, collaboration and shared responsibility. Rather than being an optional component of AMR programmes, community engagement should be recognised as a core element of equitable, context-responsive and sustainable AMR governance. Future research should empirically assess integrated community engagement approaches across One Health sectors to strengthen their implementation and inform national AMR strategies.

## Supplementary material

10.1136/bmjph-2026-004874 online supplemental table 1

## Data Availability

All data associated with this article are presented within the article.

## References

[1] Murray CJL, Ikuta KS, Sharara F (2022). Global burden of bacterial antimicrobial resistance in 2019: a systematic analysis. The Lancet.

[2] Johnson T, von Seidlein L, Cheah PY (2025). A just war on bugs? Ethical differences between antimalarial resistance and antibacterial resistance. BMJ Glob Health.

[3] Kariuki S (2024). Global burden of antimicrobial resistance and forecasts to 2050. The Lancet.

[4] O’Neill J (2016). Tackling drug-resistance infections globally: final report and recommendations. https://amr-review.org/sites/default/files/160518_Final%20paper_with%20cover.pdf.

[5] Denyer Willis L, Chandler C (2019). Quick fix for care, productivity, hygiene and inequality: reframing the entrenched problem of antibiotic overuse. BMJ Glob Health.

[6] Al Masud A, Lahiru Walpola R, Anam M (2026). Social and commercial determinants of inappropriate antibiotic use in resource-constrained settings: A social-ecological system framework analysis. Res Social Adm Pharm.

[7] Parajuli A, Garbovan L, Bhattarai B (2024). Exploring community insights on antimicrobial resistance in Nepal: a formative qualitative study. BMC Health Serv Res.

[8] Cohn J, Mendelson M, Kanj SS (2024). Accelerating antibiotic access and stewardship: a new model to safeguard public health. Lancet Infect Dis.

[9] Otaigbe II (2025). Mitigating inequitable access to appropriate antibiotics in low- and middle-income countries. JAC Antimicrob Resist.

[10] Cegielski JP, Kurbatova E, van der Walt M (2016). Multidrug-Resistant Tuberculosis Treatment Outcomes in Relation to Treatment and Initial Versus Acquired Second-Line Drug Resistance. Clin Infect Dis.

[11] Pandey S, Doo H, Keum GB (2024). Antibiotic resistance in livestock, environment and humans: One Health perspective. J Anim Sci Technol.

[12] Safdar N, Saleem S, Salman M (2025). Economic burden of antimicrobial resistance on patients in Pakistan. Front Public Health.

[13] Aslam B, Asghar R, Muzammil S (2024). AMR and Sustainable Development Goals: at a crossroads. Global Health.

[14] Ferdinand AS, Coppo MJC, Howden BP (2023). Tackling antimicrobial resistance by integrating One Health and the Sustainable Development Goals. BMC Glob Public Health.

[15] Group WB (2025). Global health expenditure database, world health organization; global health expenditure database, world health organization.

[16] Do NTT, Vu HTL, Nguyen CTK (2021). Community-based antibiotic access and use in six low-income and middle-income countries: a mixed-method approach. Lancet Glob Health.

[17] Sulis G, Adam P, Nafade V (2020). Antibiotic prescription practices in primary care in low- and middle-income countries: A systematic review and meta-analysis. PLoS Med.

[18] Thapa P, Tandan M, Bhandari B (2026). Antibiotic dispensing practices and determinants among informal healthcare providers in low- and middle-income countries: a mixed-methods scoping review. BMJ Glob Health.

[19] Vats P, Kaur UJ, Rishi P (2022). Heavy metal-induced selection and proliferation of antibiotic resistance: A review. J Appl Microbiol.

[20] Cocker D, Mwapasa T, Grabic R (2025). Environmental hazards from pollution of antibiotics and resistance-driving chemicals in an urban river network from Malawi. NPJ Antimicrob Resist.

[21] Byarugaba I, Nabatanzi A, Muhumuza E (2025). Impact of heavy metals on antibiotic resistance of Escherichia coli from slum wastewater in Kawempe division, Kampala district, Uganda: a case study. BMC Microbiol.

[22] Pal C, Bengtsson-Palme J, Kristiansson E (2015). Co-occurrence of resistance genes to antibiotics, biocides and metals reveals novel insights into their co-selection potential. BMC Genomics.

[23] Kakkar AK, Shafiq N, Singh G (2020). Antimicrobial Stewardship Programs in Resource Constrained Environments: Understanding and Addressing the Need of the Systems. Front Public Health.

[24] Li J, Zhou P, Wang J (2023). Worldwide dispensing of non-prescription antibiotics in community pharmacies and associated factors: a mixed-methods systematic review. Lancet Infect Dis.

[25] Adhikari B, Pokharel S, Raut S (2021). Why do people purchase antibiotics over-the-counter? A qualitative study with patients, clinicians and dispensers in central, eastern and western Nepal. BMJ Glob Health.

[26] Mitchell J, Hawkings H, Latham S (2023). Addressing antimicrobial resistance through community engagement: a framework for developing contextually relevant and impactful behaviour change interventions. JAC Antimicrob Resist.

[27] Hosain M, Kabir SML, Kamal M (2021). Antimicrobial uses for livestock production in developing countries. Vet World.

[28] Bhandari G, Zomer P, Atreya K (2019). Pesticide residues in Nepalese vegetables and potential health risks. Environ Res.

[29] Pham Y, Wozniak TM (2025). Systems thinking to understand the complexity of antimicrobial resistance across One Health: A systematic review of current approaches. One Health.

[30] Adhikari S, Rijal KR, Parker DM (2025). Stakeholder analysis for “One Health” approach to tackle antimicrobial resistance. BMJ Glob Health.

[31] Caudell MA, Dorado-Garcia A, Eckford S (2020). Towards a bottom-up understanding of antimicrobial use and resistance on the farm: A knowledge, attitudes, and practices survey across livestock systems in five African countries. PLoS ONE.

[32] Mehtarpour M, Najafi Z, Jaafaripooyan E (2025). Sociocultural determinants of antimicrobial resistance in Iran: a qualitative study. BMC Public Health.

[33] Mathew P, Chandy SJ, Ranjalkar J (2024). Community engagement to mitigate antimicrobial resistance in low-and middle-income countries - an essential strategy for implementation of national action plans on AMR. Lancet Reg Health Southeast Asia.

[34] Ahmad R, Zhu NJ, Leather AJM (2019). Strengthening strategic management approaches to address antimicrobial resistance in global human health: a scoping review. BMJ Glob Health.

[35] Mitchell J, Cooke P, Baral S (2019). The *values* and *principles* underpinning community engagement approaches to tackling antimicrobial resistance (AMR). Glob Health Action.

[36] World Health Organization (2018). Primary health care: closing the gap between public health and primary care through integration. https://iris.who.int/items/28af1691-2db5-451f-8a2d-477df8eb5191.

[37] Mitchell J, Cooke P, Ahorlu C (2022). Community engagement: The key to tackling Antimicrobial Resistance (AMR) across a One Health context?. Glob Public Health.

[38] Musoke D, Namata C, Lubega GB (2021). Access, use and disposal of antimicrobials among humans and animals in Wakiso district, Uganda: a qualitative study. J Pharm Policy Pract.

[39] Auta A, Hadi MA, Oga E (2019). Global access to antibiotics without prescription in community pharmacies: A systematic review and meta-analysis. J Infect.

[40] Ndaki PM, Mwanga JR, Mushi MF (2025). Drivers of inappropriate use of antibiotics among community members in low- and middle-income countries: a systematic review of qualitative studies. BMC Public Health.

[41] Maduko W, Olamijuwon E, Kesby M (2025). Public-targeted interventions addressing antimicrobial resistance and antibiotic use in Sub-Saharan Africa: a scoping review. BMJ Glob Health.

[42] King R, Hicks J, Rassi C (2020). A process for developing a sustainable and scalable approach to community engagement: community dialogue approach for addressing the drivers of antibiotic resistance in Bangladesh. BMC Public Health.

[43] Roulette CJ, Caudell MA, Roulette JW (2017). A two-month follow-up evaluation testing interventions to limit the emergence and spread of antimicrobial resistant bacteria among Maasai of northern Tanzania. BMC Infect Dis.

[44] Levin AS, Costa SF, Razzolini MTP (2023). Launch of the São Paulo Wellcome Trust-funded multidisciplinary research program on optimising antimicrobial use in highly populated urban environments. Lancet Reg Health Am.

[45] Irawati L, Alrasheedy AA, Hassali MA (2019). Low-income community knowledge, attitudes and perceptions regarding antibiotics and antibiotic resistance in Jelutong District, Penang, Malaysia: a qualitative study. BMC Public Health.

[46] Lemma M, Alemu B, Amenu K (2025). Enhancing community awareness of antimicrobial use and resistance through community conversations in rural Ethiopia. One Health Outlook.

[47] Rware H, Monica KK, Idah M (2024). Examining antibiotic use in Kenya: farmers’ knowledge and practices in addressing antibiotic resistance. CABI Agric Biosci.

[48] Tumwine C, Kiggundu R, Lwaigale F (2025). Strengthening Community Antimicrobial Stewardship in Africa: A Systematic Review of the Roles, Challenges, and Opportunities of Community Health and Animal Health Workers. Wellcome Open Res.

[49] Mishra SR, Joshi B, Poudyal Y (2022). Epistemic indebtedness: Do we owe to epistemic enterprises?. Journal of Global Health Economics and Policy.

[50] Poudyal Y, Aryal N, Rajbhandari A (2025). Over the counter use of topical corticosteroid for skin conditions among patients before attending skin specialist clinic in Nepal: A qualitative study. PLOS Glob Public Health.

[51] Pokharel S, Adhikari B (2020). Antimicrobial resistance and over the counter use of drugs in Nepal. J Glob Health.

[52] Jones III D (2007). The Hippocratic Oath III: Do no harm, withdrawal of treatment, and the mental capacity act. CMQ.

[53] Yen CF, Cutrell JB (2021). Antimicrobial ethicists: Making ethics explicit in antimicrobial stewardship. Antimicrob Steward Healthc Epidemiol.

[54] Rosa WE, Pandey S, Wisniewski R (2025). Antimicrobials in serious illness and end-of-life care: lifting the veil of silence. Lancet Infect Dis.

[55] Pokharel S, Adhikari B, Johnson T (2024). Interventions to address antimicrobial resistance: an ethical analysis of key tensions and how they apply in low- income and middle-income countries. BMJ Glob Health.

[56] Pulcini C, Collignon P (2017). Hospital antimicrobial stewardship: the way forward. Lancet Infect Dis.

[57] Cassini A, Tacconelli E (2023). Potential for improvement in governance and national action plans to overcome antimicrobial resistance. Lancet Infect Dis.

[58] Van den Bruel A (2025). Rapid diagnostics to improve antibiotic prescribing in children: one piece of a much larger jigsaw. Lancet Reg Health Eur.

[59] Hicks JP, Latham SM, Huque R (2021). Antibiotic practices among household members and their domestic animals within rural communities in Cumilla district, Bangladesh: a cross-sectional survey. BMC Public Health.

[60] Das P, Horton R (2016). Antibiotics: achieving the balance between access and excess. The Lancet.

[61] Mendelson M, Røttingen J-A, Gopinathan U (2016). Maximising access to achieve appropriate human antimicrobial use in low-income and middle-income countries. The Lancet.

[62] Anderson M, Ljungqvist G, Kessel R (2024). The socioeconomic drivers and impacts of antimicrobial resistance: implications for policy and research.

[63] Pearson M, Chandler C (2019). Knowing antmicrobial resistance in practice: a multi-country qualitative study with human and animal healthcare professionals. Glob Health Action.

[64] Parsonage B, Hagglund PK, Keogh L (2017). Control of Antimicrobial Resistance Requires an Ethical Approach. Front Microbiol.

[65] Khan M, Rahman-Shepherd A, Bory S (2022). How conflicts of interest hinder effective regulation of healthcare: an analysis of antimicrobial use regulation in Cambodia, Indonesia and Pakistan. BMJ Glob Health.

[66] Harun MGD, Sumon SA, Hasan I (2024). Barriers, facilitators, perceptions and impact of interventions in implementing antimicrobial stewardship programs in hospitals of low-middle and middle countries: a scoping review. Antimicrob Resist Infect Control.

[67] Rynkiewich K (2022). Antimicrobial prescribing matters: the irreconcilability in moral ranking systems. Anthropol Med.

[68] Basu S, Garg S (2018). Antibiotic prescribing behavior among physicians: ethical challenges in resource-poor settings. J Med Ethics Hist Med.

[69] Liu B, Wang W, Deng Z (2023). Antibiotic governance and use on commercial and smallholder farms in eastern China. Front Vet Sci.

[70] Acosta A, Tirkaso W, Nicolli F (2025). The future of antibiotic use in livestock. Nat Commun.

[71] Ardakani Z, Aragrande M, Canali M (2024). Global antimicrobial use in livestock farming: an estimate for cattle, chickens, and pigs. Animal.

[72] Van Boeckel TP, Brower C, Gilbert M (2015). Global trends in antimicrobial use in food animals. Proc Natl Acad Sci USA.

[73] Olaru ID, Walther B, Schaumburg F (2023). Zoonotic sources and the spread of antimicrobial resistance from the perspective of low and middle-income countries. Infect Dis Poverty.

[74] Varadan SR, Chandler CIR, Weed K (2024). A just transition for antimicrobial resistance: planning for an equitable and sustainable future with antimicrobial resistance. The Lancet.

[75] Mshana SE, Sindato C, Matee MI (2021). Antimicrobial Use and Resistance in Agriculture and Food Production Systems in Africa: A Systematic Review. Antibiotics (Basel).

[76] Tiseo K, Huber L, Gilbert M (2020). Global Trends in Antimicrobial Use in Food Animals from 2017 to 2030. Antibiotics (Basel).

[77] Hutchings MI, Truman AW, Wilkinson B (2019). Antibiotics: past, present and future. Curr Opin Microbiol.

[78] Bwanali AN, Lubanga AF, Kondowe S (2025). Trends and patterns of antimicrobial resistance among common pathogens isolated from adult bloodstream and urinary tract infections in public health facilities in Malawi, 2020–2024. BMC Infect Dis.

[79] Sharma S, Satyaseela MP, Burri RR (2025). Combating Antimicrobial Resistance: Role of Key Stakeholders with Focus on the Pharmaceutical Sector. Pharm Med.

[80] Adhikari B, Pell C, Cheah PY (2020). Community engagement and ethical global health research. Glob Bioeth.

[81] Singh S, Charani E, Devi S (2021). A road-map for addressing antimicrobial resistance in low- and middle-income countries: lessons learnt from the public private participation and co-designed antimicrobial stewardship programme in the State of Kerala, India. Antimicrob Resist Infect Control.

[82] Gilham EL, Pearce-Smith N, Carter V (2024). Assessment of global antimicrobial resistance campaigns conducted to improve public awareness and antimicrobial use behaviours: a rapid systematic review. BMC Public Health.

[83] World Health Organization (2015). Global action plan on antimicrobial resistance. https://iris.who.int/handle/10665/193736.

[84] Acharya S, Mishra SR, Seidlein L (2025). Reimagining primary health care: a historical and contemporary review of community-based care models and innovations. In Review.

[85] Poomchaichote T, Kiatying-Angsulee N, Boonthaworn K (2024). Embedding community and public voices in co-created solutions to mitigate antimicrobial resistance (AMR) in Thailand using the “Responsive Dialogues” public engagement framework. Antimicrob Resist Infect Control.

[86] Pawson R, Tilley N (2004). Realist evaluation.

