# Supplementary material for: Community engagement strategies for antimicrobial resistance control and interventions
Source: BMJ Public Health. 2026 Aug 4;4(3):e004874. doi: 10.1136/bmjph-2026-004874 (PMC13448595; doi:10.1136/bmjph-2026-004874)
Supplement: Supplementary file 1 [file bmjph-4-3-s001.docx]

# **Supplementary Table S1**

# Community engagement strategies for antimicrobial resistance control and interventions

Sanjaya Acharya MD^1^, Thomas J Peto PhD^2,3^, Abhilasha Karkey DPhil^3,4^, Phaik Yeong Cheah PhD^2,3^, Bipin Adhikari DPhil^2,3*^

^1^Academy for Data Science and Global Health, Kathmandu, Nepal

^2^Mahidol-Oxford Tropical Medicine Research Unit, Faculty of Tropical Medicine, Mahidol University, Bangkok, Thailand

^3^Centre for Tropical Medicine and Global Health, Nuffield Department of Medicine, University of Oxford, Oxford, United Kingdom

^4^Oxford University Clinical Research Unit, Kathmandu, Nepal

*Bipin Adhikari; email: Bipin@tropmedres.ac

**Supplementary Table S1**: Community engagement strategies highlighting breadth-based and depth-based approaches

|  | **Complementary components of a diagonal community engagement strategy** | |
| --- | --- | --- |
|  | **Breadth-based approach** | **Depth-based approach** |
| Definition | Refers to strategies that aim to engage a wide range of community stakeholders to raise awareness and promote general understanding of AMR. | Focuses on targeted, in-depth engagement with specific community groups or individuals to understand and address local drivers of AMR and co-develop context-specific solutions. |
| Typical activities | Often involves mass campaigns (radio, TV health campaigns), community meetings, and public health education initiatives designed to reach a large audience. | Involves tailored interventions such as training workshops for community health workers, conducting participatory action research and co-design, community dialogues to foster deeper understanding of the topic and individual-level engagement. |
| Appropriateness | 1. National awareness campaigns 2. Population-wide communication 3. Early-stage engagement | 1. Co-designing interventions ( e.g. to equitable interventions) 2. Addressing local barriers 3. Refining implementation strategies |
| Resources | 1. National media platforms 2. Government commitment 3. Public health infrastructure | 1. Community members 2. local leaders 3. Community organizations 4. Skilled facilitators |
| Merits | 1. Can achieve wide coverage. 2. Efficiently raises awareness across large populations (e.g. hand hygiene) and preventive behavior. 3. Mass communication can be cost-effective as it can obviate the need to reach individuals. | 1. Can enhance quality of interaction. 2. Can tailor the messages/strategies for specific socio-cultural context. 3. Enhances the stakeholders’ participation and ownership. |
| Pitfalls | 1. Messages may remain generic (not able to explain in details through for example rationales and individually raised questions). 2. May result in inadequate understanding and conceptualization by the targeted audience. 3. Lack of opportunity to interact and ask questions, can reduce the actions taken towards it, including assessment of impacts. | 1. Resource/time intensive. 2. Difficult to scale. 3. Requires skilled implementers. |
| Examples* | 1. National AMR awareness and livestock guidelines (Brazil): demonstrated how a nationwide, cross-sectoral policy framework can raise awareness and set standards for antibiotic use across the livestock sector. Illustrates breadth by targeting entire industries and the public.^1^ 2. Population knowledge and awareness of antibiotic use and antimicrobial resistance: results from national household survey 2019 and changes from 2017 (Thailand): demonstrated how a nationwide, population-based assessment can capture public knowledge and awareness of antibiotic use and resistance. Illustrates breadth by informing mass communication campaigns and national policy responses.^2^ | 1. Understanding antibiotic use for pig farming (Thailand): a qualitative study: focused on specific actors (farmers, veterinarians, drug sellers) in one sector, uncovering local practices, incentives, and barriers that drive antimicrobial misuse. Demonstrates depth by engaging key groups for tailored solutions. ^3^ 2. Study of antimicrobial use in rural livestock (Kenya): examined frontline prescribers’ knowledge, attitudes, and practices in outpatient clinics, identifying precise gaps in stewardship behavior. Highlights the value of targeted interventions in specific professional groups.^4^ 3. “Responsive Dialogues” to co-create AMR solutions involving community members and key stakeholders in Thailand.^5^ |
| Impacts | 1. Population awareness 2. Broad behaviour change 3. Policy visibility | 1. Context-specific understanding 2. Equitable intervention design 3. Community awareness |
| *Breadth-based approaches typically represent the first stage of programme implementation, prioritising wide population coverage, whereas depth-based approaches prioritise intensive participatory engagement with smaller groups. The examples illustrate different engagement purposes rather than provide a comparative evaluation. | | |

# References

1.	Albernaz-Gonçalves R, Olmos Antillón G, Hötzel MJ. Waiting for markets to change me—High-stakeholders' views of antibiotic use and antibiotic resistance in pig production in Brazil. *Frontiers in Veterinary Science* 2022; **9**: 980546.

2.	Tangcharoensathien V, Chanvatik S, Kosiyaporn H, et al. Population knowledge and awareness of antibiotic use and antimicrobial resistance: results from national household survey 2019 and changes from 2017. *BMC Public Health* 2021; **21**(1): 2188.

3.	Lekagul A, Tangcharoensathien V, Liverani M, Mills A, Rushton J, Yeung S. Understanding antibiotic use for pig farming in Thailand: a qualitative study. *Antimicrobial Resistance & Infection Control* 2021; **10**(1): 3.

4.	Kaniu MW, Gitaka WR, Jain R, Munyare AN, Adam RD, Monroe-Wise A. Knowledge, attitudes, and practices regarding antimicrobial resistance and antimicrobial stewardship among healthcare workers in outpatient medical centers in Kenya: a qualitative study. *Antimicrobial Stewardship & Healthcare Epidemiology* 2025; **5**(1): e113.

5.	Poomchaichote T, Kiatying-Angsulee N, Boonthaworn K, et al. Embedding community and public voices in co-created solutions to mitigate antimicrobial resistance (AMR) in Thailand using the ‘Responsive Dialogues’ public engagement framework. *Antimicrobial Resistance & Infection Control* 2024; **13**(1): 71.
